# Posterior Fossa Sinking Skin Flap Syndrome Presenting With Orthostatic Vertigo After Decompressive Craniectomy: A Case Report

**DOI:** 10.7759/cureus.101765

**Published:** 2026-01-18

**Authors:** Ryo Matsuzaki, Yuki Sakaeyama, Shuhei Kubota, Sayaka Terazono, Nobuo Sugo

**Affiliations:** 1 Department of Neurosurgery, Faculty of Medicine, Toho University, Tokyo, JPN

**Keywords:** cranioplasty timing, posterior fossa repair, sinking skin flap syndrome, suboccipital craniectomy, ventriculoperitoneal (vp) shunt

## Abstract

Sinking skin flap syndrome (SSFS) is an uncommon complication of cranial defects and is rarely described after posterior fossa decompression. We report a case of chronic phase posterior fossa SSFS presenting with positional vertigo and vomiting, successfully treated with delayed cranioplasty. A 40-year-old man presented with subarachnoid hemorrhage caused by a ruptured vertebral artery dissecting aneurysm involving the left posterior inferior cerebellar artery (PICA). He underwent an occipital artery (OA)-PICA bypass and trapping of the aneurysm, followed by decompressive suboccipital craniectomy without immediate cranioplasty. Progressive ventriculomegaly developed, and a ventriculoperitoneal (VP) shunt (Codman Hakim, Integra Japan, Tokyo, Japan) was placed. He was later readmitted with vertigo and vomiting that worsened in the upright position and improved when supine, accompanied by a conspicuous depression and postural change of the scalp flap over the defect. Posterior fossa SSFS was diagnosed, and cranioplasty was performed, leading to rapid and sustained symptom resolution. SSFS should be considered in patients with large posterior fossa defects who develop posture-dependent symptoms, particularly when local soft-tissue support is limited; timely cranioplasty may be therapeutic.

## Introduction

Cranioplasty is performed whenever feasible after craniectomy, and its purpose extends beyond cosmetic restoration to include functional neurological recovery [1]. The so-called sinking skin flap syndrome (SSFS) is thought to result from persistent decompression, leading to alterations in cerebral blood flow and cerebrospinal fluid (CSF) dynamics [1,2]. Clinically, SSFS may present with a sunken scalp flap and posture-dependent symptoms such as orthostatic headache, dizziness/vertigo, and neurological deterioration [1,2]. However, prior reports supporting the clinical necessity of cranioplasty have largely focused on supratentorial cranial defects (above the tentorium cerebelli), with little to no discussion of infratentorial defects involving the posterior fossa (below the tentorium, surrounding the cerebellum and brainstem) [1-5]. Here, we report a case of posterior fossa SSFS that presented in the chronic phase with orthostatic vertigo after posterior fossa decompression and showed marked improvement following occipital cranioplasty.

## Case presentation

**Figure 1:**
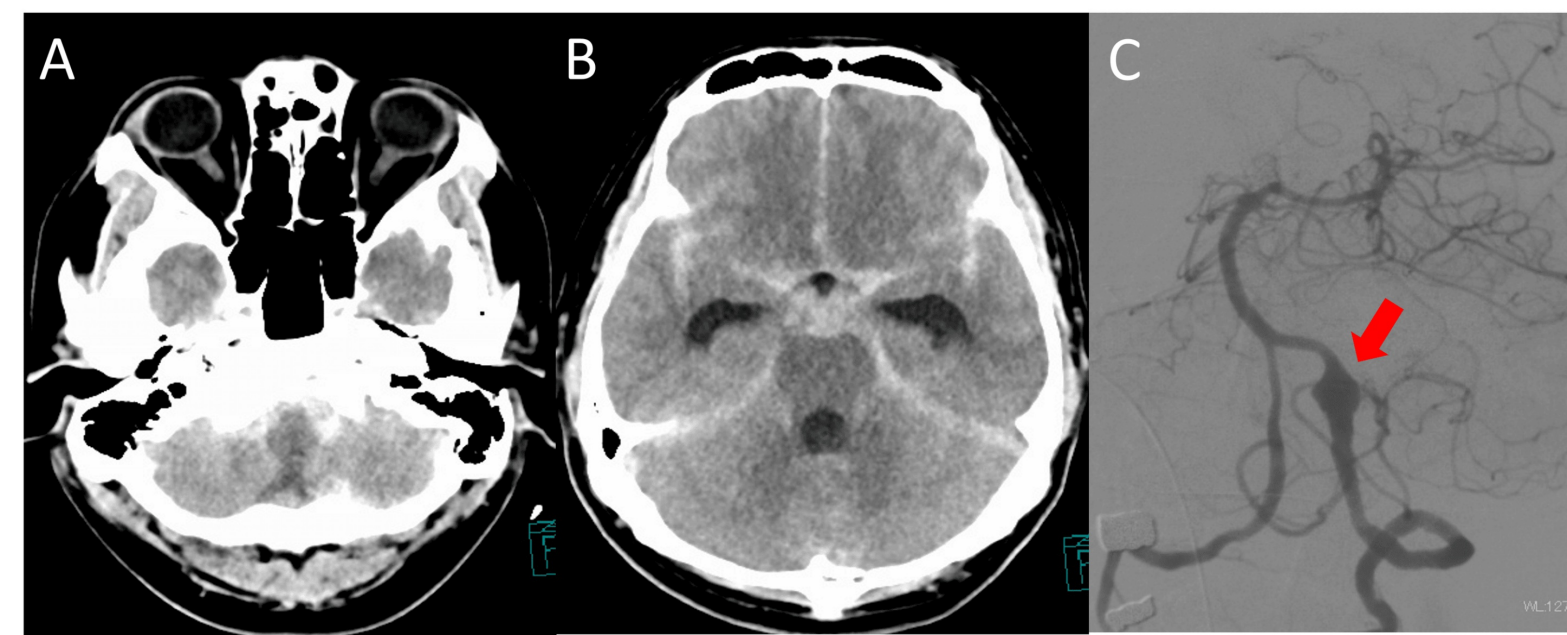
Initial imaging findings. (A) Initial non-contrast head CT demonstrating thick subarachnoid clot around the brainstem. (B) Initial CT showing subarachnoid hemorrhage within the basal cisterns. (C) Initial cerebral angiography demonstrating left vertebral artery dissection (arrow).

A 40-year-old man was transported to our emergency department after the sudden onset of severe occipital headache and impaired consciousness. On arrival, his Glasgow Coma Scale score was E2V2M4, and there was no obvious quadriparesis [6]. Initial CT demonstrated diffuse subarachnoid hemorrhage distributed both supratentorially and infratentorially, corresponding to Fisher group 3, with particularly thick clot accumulation around the medulla oblongata (Figures 1A-1B). Catheter angiography revealed a left vertebral artery dissecting aneurysm (Figure 1C).

**Figure 2:**
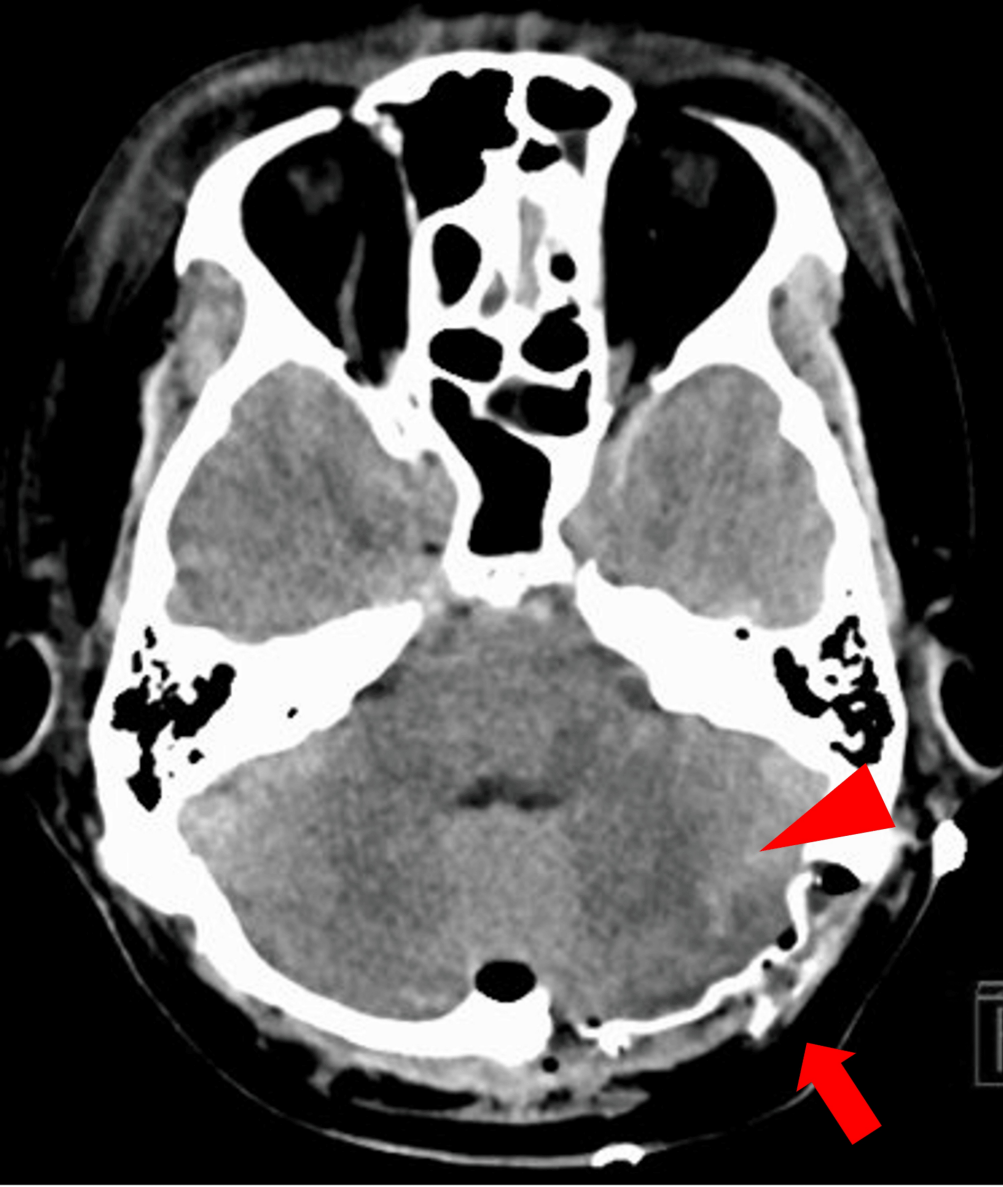
Postoperative CT after aneurysm trapping and posterior fossa decompression. Postoperative non-contrast head CT showing decompressive craniectomy without bone flap replacement (arrow). Suspected infarction in the left posterior inferior cerebellar artery (PICA) territory is noted (arrowhead).

On day 5 after onset, the patient underwent a left occipital artery (OA)-posterior inferior cerebellar artery (PICA) bypass and trapping of the ruptured aneurysm. A left posterior fossa decompressive state was intentionally maintained, and cranioplasty was not performed (Figure 2). Postoperative CT suggested ischemia in the left cerebellar hemisphere and the left medial medulla, which was confirmed on MRI on postoperative day 4. The patient was extubated on the same day. He developed complete right hemiplegia attributable to a left medial medullary infarction, without apparent cerebellar signs. The hemiparesis gradually improved to a manual muscle test (MMT) grade of 3 [7].

**Figure 3:**
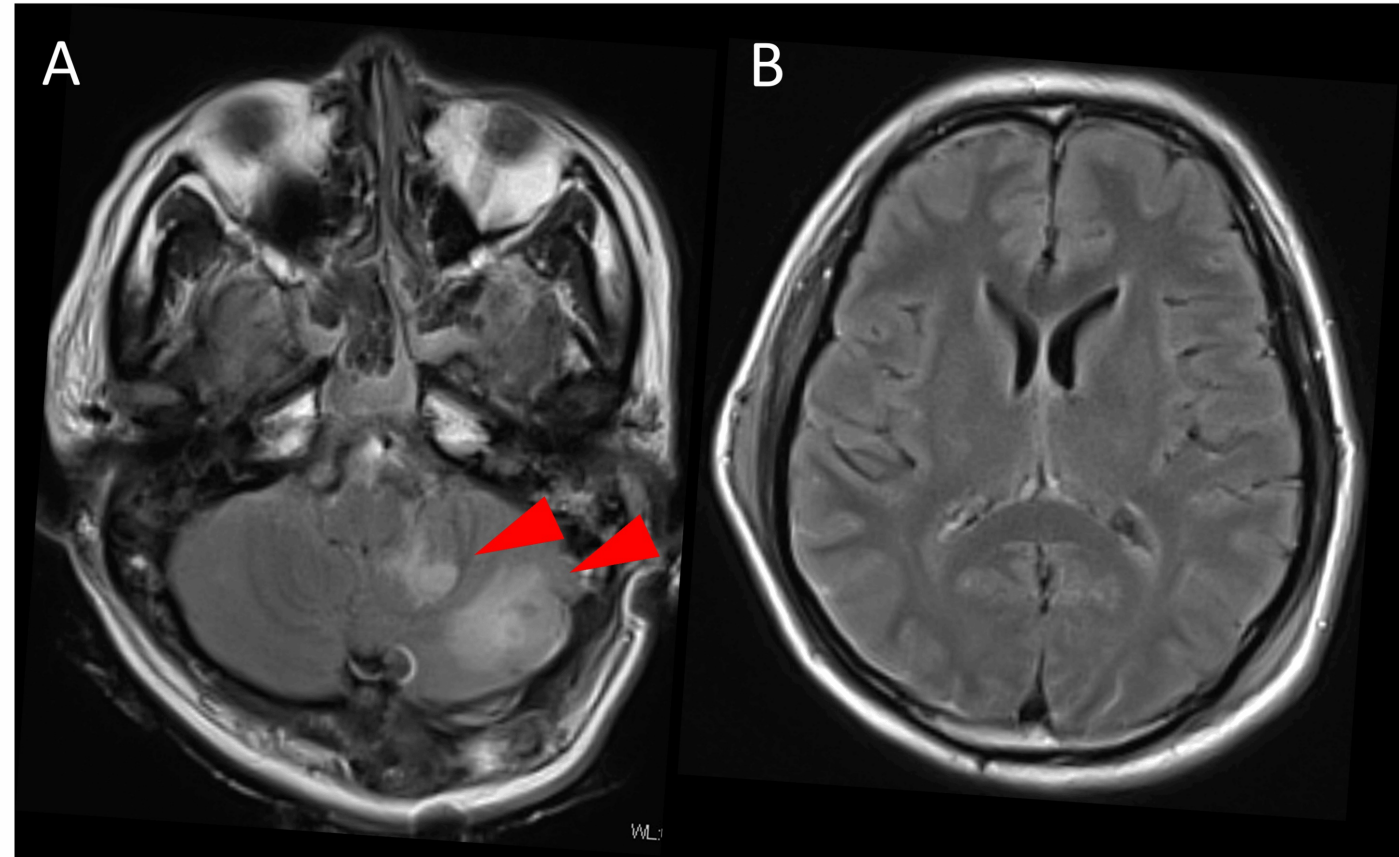
Postoperative ischemic changes and ventricular size. (A) Follow-up CT showing a well-demarcated infarction in the left posterior inferior cerebellar artery (PICA) territory (arrowheads). (B) No ventricular enlargement is observed.

On day 43 after onset, a right frontal horn ventriculoperitoneal (VP) shunt (Codman Hakim, Integra Japan, Tokyo, Japan) was placed for normal pressure hydrocephalus. The initial valve setting was 180 mmH_2_O. He was transferred to a rehabilitation hospital without cranioplasty (Figures 3A-3B). His right hemiparesis further improved to MMT grade 4, and he was discharged from the rehabilitation hospital four months after onset. 

Fourteen months after onset, he was readmitted because of vertigo and vomiting on standing. Symptoms improved after changing the shunt valve setting from 200 mmH_2_O to 120 mmH_2_O, and he was transferred back to a rehabilitation hospital. 

**Figure 4:**
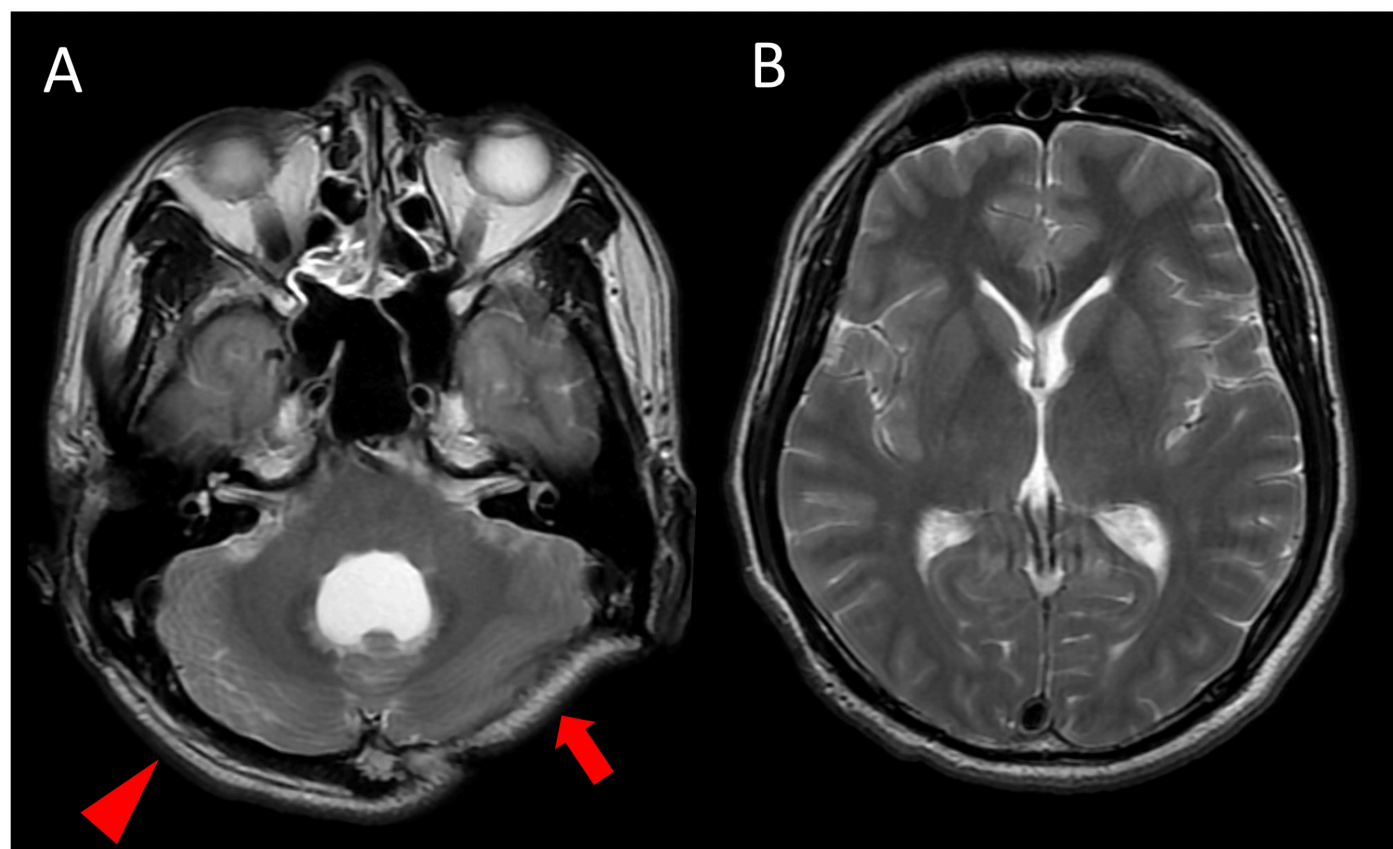
CT at readmission with orthostatic symptoms. (A) CT demonstrating enlargement of the fourth ventricle. Compared with the contralateral side (arrowhead), the skin flap on the affected side appears atrophic (arrow). (B) No enlargement of the lateral ventricles is observed.

Fifteen months after onset, he was readmitted again due to recurrent orthostatic vertigo at the rehabilitation hospital. His body mass index (BMI) had decreased from 24.8 kg/m^2^ at the time of subarachnoid hemorrhage onset to 21.2 kg/m^2^ at this readmission. On examination, he was alert. Vertigo resolved in the supine position but recurred approximately one minute after elevating his trunk, accompanied by vomiting and dysphagia. Head CT demonstrated mild hydrocephalus and enlargement of the fourth ventricle, leading to a diagnosis of shunt malfunction (Figures 4A-4B). On axial CT, the fourth ventricle measured 23 × 28 mm (anteroposterior × transverse), and the Evans index was 24%. The posterior fossa decompression site appeared bulging, consistent with increased intracranial pressure (ICP). 

**Figure 5:**
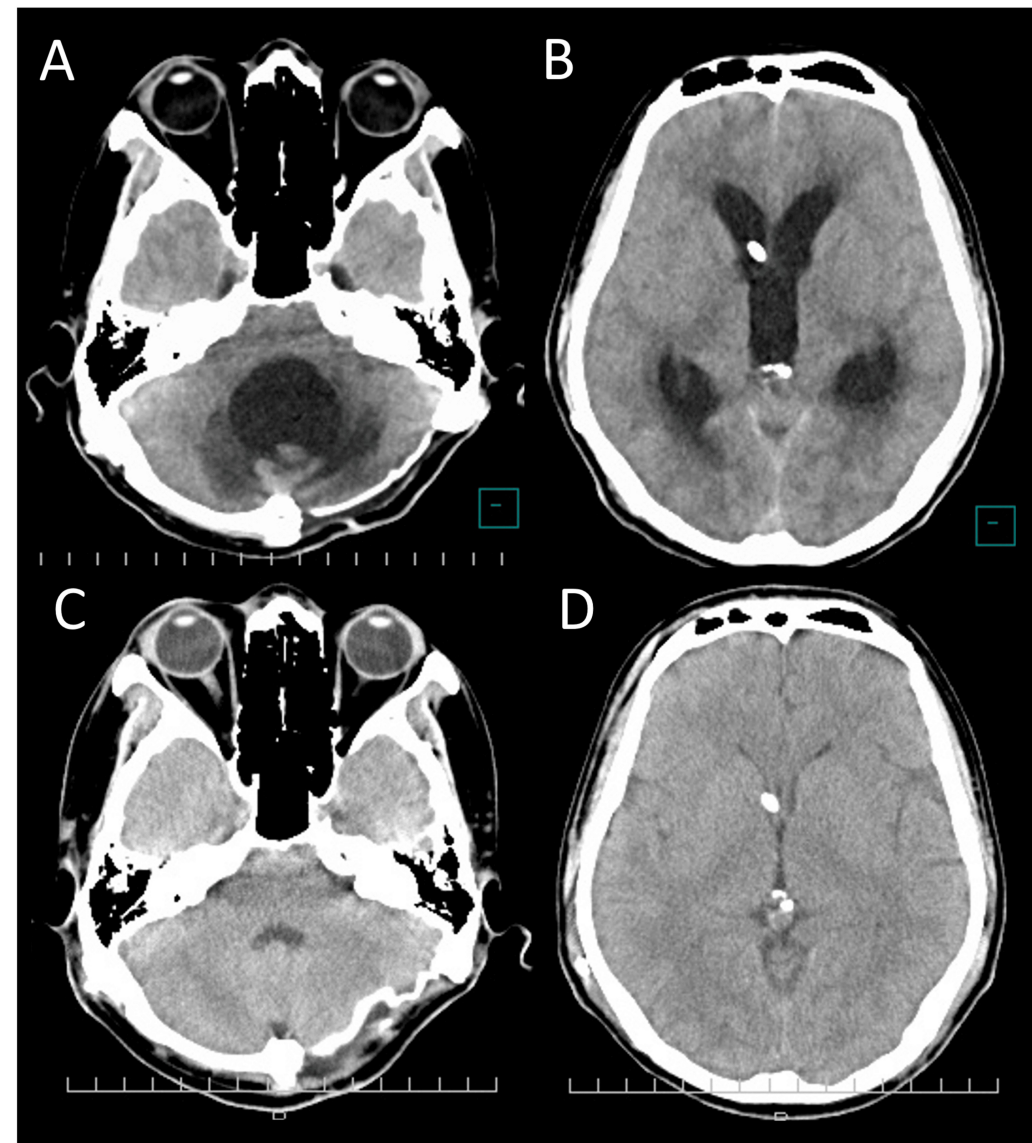
Serial CT scans during shunt valve adjustments. (A, B) Ventricular enlargement is present. (C, D) Ventricular size is reduced following shunt pressure adjustment, consistent with overdrainage.

After admission, adjustment of the shunt valve resulted in a rapid reduction of ventricular size, leading to overdrainage; despite multiple attempts at valve reprogramming, stable control was difficult (Figures 5A-5D). On axial CT, the fourth ventricle size fluctuated markedly during valve reprogramming (32 × 39 mm in Figure 5A vs. 9 × 25 mm in Figure 5C; anteroposterior × transverse), and the Evans index changed from 33% (Figure 5B) to 25% (Figure 5D). However, the orthostatic symptoms persisted despite these radiographic changes. During this period, the posterior fossa decompression site became progressively sunken. On physical examination, the skin over the decompressive defect was extremely thin, and arterial pulsation was palpable. The flap became more depressed with head elevation and appeared more tense in the supine position. Taken together, the posture-dependent symptoms and the position-dependent sunken posterior fossa flap supported the diagnosis of posterior fossa SSFS. 

**Figure 6:**
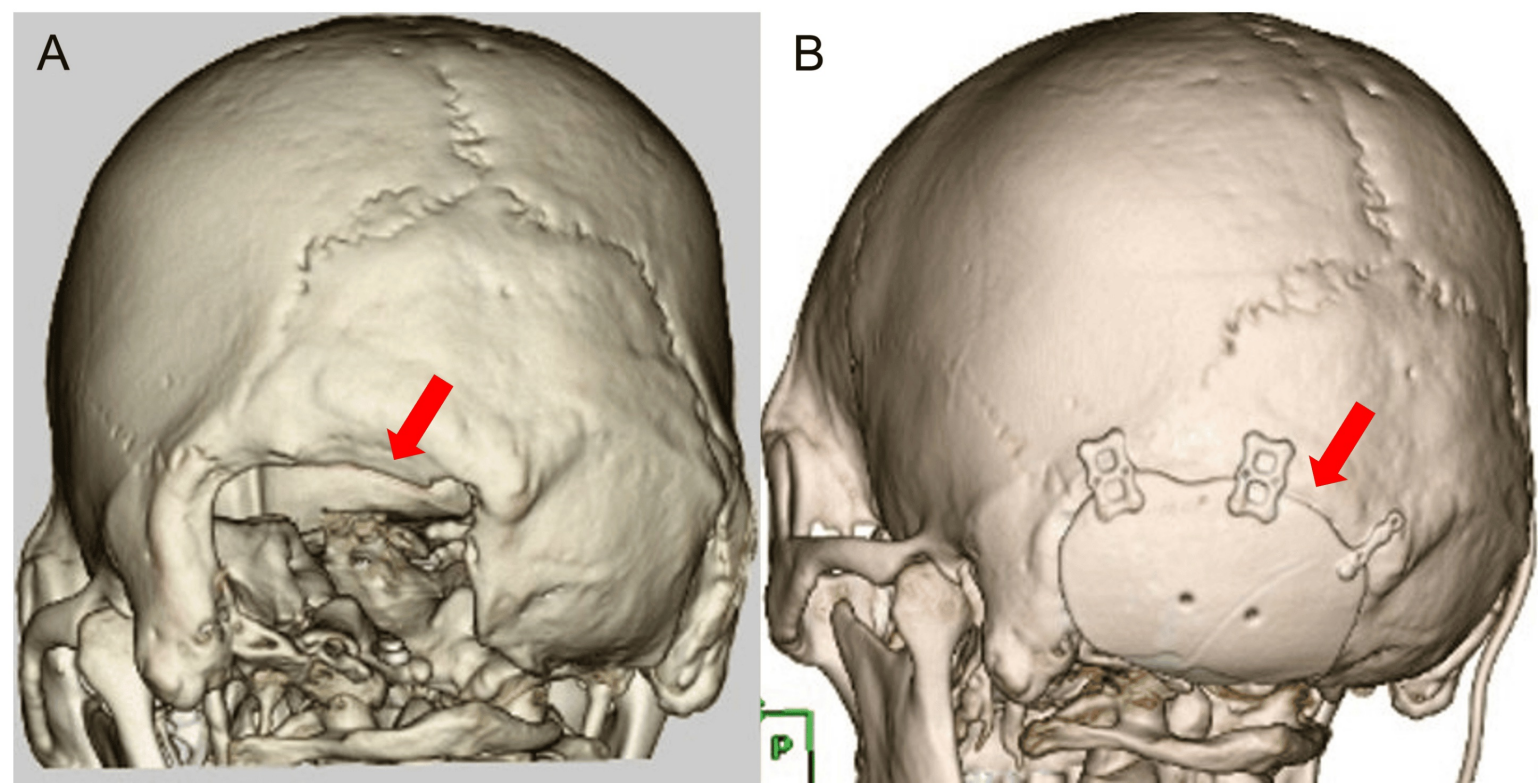
Preoperative 3D bone imaging and postoperative reconstruction. (A) Preoperative 3D CT of the skull demonstrating the left posterior fossa defect (arrow). (B) Postoperative CT after posterior fossa cranioplasty with a synthetic implant secured using titanium plates (arrow).

Given the difficulty in shunt adjustment and the presumed mechanism - progressive thinning of the skin flap and occipital musculature at the posterior fossa defect together with brain atrophy - posterior fossa cranioplasty was performed. Postoperatively, the orthostatic vertigo and dysphagia resolved completely (Figure 6). He was discharged home with a modified Rankin Scale score of 0 [8]. At the seven-year follow-up, he remained free of recurrent orthostatic vertigo or dysphagia, was fully independent in activities of daily living, had returned to work, and was tolerating a normal diet consistency. No additional neurosurgical interventions were required, and there were no postoperative complications such as CSF leakage.

## Discussion

General background on SSFS 

SSFS, also known as syndrome of the trephined, is a rare complication of large craniectomy characterized by new neurological deficits that typically worsen in the upright position and improve after cranioplasty [9]. First described by Grant in 1939 and later coined “sinking skin flap syndrome” by Yamaura in 1977, this condition can manifest weeks to months after decompressive surgery [9,10]. Patients may develop headaches, dizziness, cognitive or mood changes, and motor deficits due to the skull defect - and importantly, these symptoms often reverse after restoration of the cranial vault [9]. Although uncommon, SSFS is clinically significant because it is a treatable cause of neurological deterioration: timely cranioplasty can normalize ICP dynamics and cerebral blood flow, leading to dramatic neurological improvement rather than serving merely a cosmetic purpose [2,9].

Posterior fossa SSFS: rarity and anatomical considerations 

SSFS has been reported almost exclusively in patients with large supratentorial (hemispheric) craniectomies - for example, after fronto-temporo-parietal decompression for trauma or stroke [11]. SSFS arising in the infratentorial compartment is exceedingly rare and, to our knowledge, has been seldom reported. In the posterior fossa, the brain is supported by the surrounding skull and tentorium, potentially reducing the gravitational “sag” seen with hemispheric defects [12]. A recent study by Yoshida et al. found that postural brain shifts after craniectomy were significantly larger with supratentorial defects than with infratentorial ones [12]. This may explain why virtually all documented SSFS cases have involved supratentorial bone defects, and no prior cases in the posterior fossa have been clearly documented in the literature [12]. The present case is thus unique as an apparently first-reported instance of SSFS in a posterior fossa craniectomy, expanding the anatomical locations in which this syndrome can occur.

Pathophysiology: ICP gradient and CSF dynamics 

The pathophysiology of SSFS revolves around the loss of the normal cranial “closed box” environment [13]. After a decompressive craniectomy, the rigid skull can no longer contain or counteract changes in ICP [13]. Atmospheric pressure and gravity thereby have a direct impact on intracranial contents through the defect [13]. In the upright position, venous pooling and CSF redistribution cause ICP to drop; with a large skull defect, the relatively higher atmospheric pressure can push the scalp inward, “sinking” the skin flap and underlying brain [14]. This creates a pressure gradient that compresses the cortex and can even lead to a dangerous downward displacement of the brain (so-called paradoxical herniation) in extreme cases [13]. Dujovny et al. postulated that three factors act in concert: reduced cerebral perfusion, altered CSF hydrodynamics, and direct external pressure on the brain all contribute to the syndrome [3]. In summary, an unprotected brain is susceptible to external pressure and volume shifts, especially when upright, leading to reversible neurological dysfunction until the cranial defect is closed.

Literature review: cranioplasty effects on neurological function 

Multiple reports have demonstrated that cranioplasty leads to objective physiological and clinical improvements in patients with SSFS [13]. In a systematic review of 205 cases, Halani et al. found that restoring the skull defect resulted in a significant increase in cerebral blood flow in the affected hemisphere [2]. Many patients also showed improved perfusion in the contralateral hemisphere and overall neurological gains after cranioplasty [2]. This supports the concept that sunken flap syndrome is at least partly mediated by hypoperfusion of the brain, which cranioplasty can reverse [2]. Dujovny et al. provided direct evidence of CSF flow normalization after cranioplasty using phase-contrast MRI in an adult syndrome of trephined patient [3]. They reported that venous outflow improved and craniocaudal CSF systolic flow velocity doubled after the bone defect was closed [3]. These hemodynamic changes reflect a restoration of normal craniospinal compliance once the rigid skull is re-established [3]. Clinically, nearly all cases of SSFS show at least partial neurological recovery post-cranioplasty, often within days, from improved level of consciousness and cognitive function to resolution of focal deficits [2]. Thus, modern literature underscores that cranioplasty is not purely cosmetic but rather therapeutic as it corrects the pathophysiological state of the “open” cranium and allows the brain to function in a normalized pressure environment [13].

Risk factors for developing SSFS 

Not every craniectomy patient develops SSFS, and researchers have sought to identify what risk factors make SSFS more likely [12]. Broadly, any factor that lowers the resistance or support at the cranial defect can predispose the patient to a sunken flap and brain shift [12]. Yoshida et al. (2022) noted that patients with a very large skull defect (greater area of bone removal) are at higher risk, as a larger “open window” in the skull permits more brain deformation [12]. A long interval between craniectomy and cranioplasty is also associated with SSFS, presumably because the scalp and dura flaps undergo scar contracture and atrophy over time, becoming thinner and less tensile [12]. Significant brain atrophy or volume loss (for instance, from infarction or underlying injury) further reduces intracranial volume, creating extra space that promotes sinking [12]. Another important risk factor is CSF hypovolemia - patients with CSF diversion shunts or excessive CSF drainage are prone to intracranial hypotension, allowing the atmosphere to press in on the brain more readily [15]. Jeyaraj documented that neurological deterioration after craniectomy can be exacerbated by a VP shunt siphoning CSF, and that subsequent cranioplasty led to rapid reversal of deficits [1]. Finally, the presence of intracranial air (pneumocephalus) in the early post-op period is a transient risk factor that can worsen pressure gradients - air can rise to the craniectomy site and exert pressure on the brain or stretch bridging veins, especially when the patient sits or stands [12]. In summary, a wide bone defect, a thin/atrophic scalp flap, brain parenchymal loss, and low CSF pressure are all conditions that tip the balance toward the sinking skin flap phenomenon. Our patient, unfortunately, had multiple such factors, which likely acted in synergy to produce SSFS in an unusual location [1,12,15].

Proposed mechanisms in the present case 

Several predisposing features in this case help explain why a posterior fossa craniectomy led to SSFS. First, the patient’s suboccipital craniectomy created a bony defect in the occipital region, measuring approximately 65 × 50 mm in maximal dimensions (Figure 6). Although smaller than a typical hemicraniectomy, this defect was sufficient to disrupt normal cranial pressure equilibrium. Second, the patient experienced a lateral medullary (PICA territory) infarction in the postoperative course, resulting in focal cerebellar atrophy. This atrophy effectively increased the extracerebellar space under the craniectomy, promoting internal pressure drop and brain shift. Third, over the interval of healing, the patient’s muscle and scalp flap became very thin - partly due to disuse atrophy of the neck muscles and partly due to the patient’s nutritional status/weight loss (his BMI dropped from 24.8 kg/m^2^ to 21.2 kg/m^2^, reflecting loss of subcutaneous fat and muscle mass). A thin, lax scalp flap offers little resistance to inward collapse at the defect. Perhaps most importantly, the patient had a VP shunt placed for hydrocephalus, which likely created chronically low CSF pressure around the brain. When the patient was upright, gravitational siphoning by the shunt would further decrease intracranial CSF volume, exacerbating the negative pressure gradient at the craniectomy site. A similar mechanism may be relevant after posterior fossa decompression for Chiari malformation, where a suboccipital defect combined with altered CSF dynamics (e.g., CSF hypovolemia from leakage or overdrainage and/or CSF diversion) can predispose to posture-dependent flap deformation and brainstem/cerebellar traction, resulting in orthostatic symptoms [16]. All of these factors combined to produce a marked postural syndrome: on standing, the patient’s cerebellum would sag and CSF would shift, causing the scalp at the defect to retract inward initially and then bulge outward with a transmitted fluid wave. He experienced immediate vertigo and vomiting in this position as the brainstem/cerebellum was distorted and perfusion compromised. When he lay flat, ICP equalized and the brain shift reduced, leading to near-complete symptom relief. This classic dependence of symptoms on posture strongly indicated SSFS. Once the patient underwent cranioplasty (repairing the skull defect with a custom plate), his intracranial compartment was again enclosed and supported. Consequently, his symptoms resolved completely - a definitive outcome confirming the diagnosis of SSFS.

Upright imaging for diagnosis of SSFS 

Diagnosing SSFS can be challenging when radiological signs are subtle. Traditional supine CT or MRI often shows a sunken scalp flap, but the degree of brain shift may not be obvious without the gravitational effect [12]. A novel solution to this diagnostic gap is upright neuroimaging [12]. Recently, Yoshida et al. demonstrated the utility of an upright CT scanner in a case of supratentorial SSFS [12]. In that patient, conventional supine CT appeared relatively normal, but upright CT revealed a dramatic downward shift of the brain and midline structures when the patient sat up, corresponding to his neurological deterioration [12]. After cranioplasty, repeat upright CT showed that the positional brain shift had resolved, paralleling the patient’s recovery [12]. This report highlights that upright imaging can directly visualize the positional component of the syndrome, which is otherwise inferred only clinically [12]. In our case, an upright CT was not obtained, but it would likely have shown brainstem descent or additional crowding at the foramen magnum upon standing. Going forward, the availability of upright CT may improve recognition of SSFS, especially in equivocal cases. Clinicians should be aware that if a post-craniectomy patient’s symptoms fluctuate with posture, obtaining imaging in the upright position (when feasible) could objectively demonstrate the sinking and aid in diagnosis. This tool may be particularly useful in underrecognized scenarios like posterior fossa SSFS, potentially guiding timely intervention.

Limitations 

ICP monitoring and standardized symptom severity scales were not obtained in this case, which limits objective quantification of the relationship between CSF dynamics, flap deformation, and symptom severity. 

## Conclusions

This case demonstrates that SSFS is not limited to large hemispheric craniectomies; it can also arise after posterior fossa procedures when local support is insufficient. Clinicians should therefore maintain a high index of suspicion in any post-craniectomy patient with otherwise unexplained neurological deterioration, particularly when symptoms improve with recumbency, regardless of defect size or location. In such settings, early cranioplasty should be considered as definitive therapy. Our patient’s complete recovery after occipital cranioplasty highlights that timely restoration of the cranial vault can rapidly normalize intracranial physiology and reverse symptoms. Overall, modern evidence supports cranioplasty as a therapeutic intervention for the syndrome, rather than a purely cosmetic procedure, and prompt treatment may prevent avoidable morbidity in this potentially reversible condition. 

## References

[1] Jeyaraj P (2015). Importance of early cranioplasty in reversing the “syndrome of the trephine/motor trephine syndrome/sinking skin flap syndrome”. J Maxillofac Oral Surg.

[2] Halani SH, Chu JK, Malcolm JG (2017). Effects of cranioplasty on cerebral blood flow following decompressive craniectomy: a systematic review of the literature. Neurosurgery.

[3] Dujovny M, Fernandez P, Alperin N, Betz W, Misra M, Mafee M (1997). Post-cranioplasty cerebrospinal fluid hydrodynamic changes: magnetic resonance imaging quantitative analysis. Neurol Res.

[4] Watanabe J, Maruya J, Nishimaki K (2016). Sinking skin flap syndrome after unilateral cranioplasty and ventriculoperitoneal shunt in a patient with bilateral decompressive craniectomy. Interdiscip Neurosurg.

[5] Sarov M, Guichard JP, Chibarro S (2010). Sinking skin flap syndrome and paradoxical herniation after hemicraniectomy for malignant hemispheric infarction. Stroke.

[6] Teasdale G, Jennett B (1974). Assessment of coma and impaired consciousness. A practical scale. Lancet.

[7] Kendall FP, McCreary EK, Provance PG (2005). Muscles: Testing and Function With Posture and Pain. https://www.google.co.in/books/edition/Muscles_Testing_and_Function/2YZ6zgEACAAJ?hl=en.

[8] van Swieten JC, Koudstaal PJ, Visser MC, Schouten HJ, van Gijn J (1988). Interobserver agreement for the assessment of handicap in stroke patients. Stroke.

[9] Yamaura A, Makino H (1977). Neurological deficits in the presence of the sinking skin flap following decompressive craniectomy. Neurol Med Chir (Tokyo).

[10] Grant FC, Norcross NC (1939). Repair of cranial defects by cranioplasty. Ann Surg.

[11] Kim H, Yang HS, Lee GS (2020). A case of “sinking skin flap syndrome” in vegetative state patient. Korean J Neurotrauma.

[12] Yoshida K, Toda M, Yamada Y (2022). Cranial defect and pneumocephalus are associated with significant postneurosurgical positional brain shift: evaluation using upright computed tomography. Sci Rep.

[13] Akins PT, Guppy KH (2008). Sinking skin flaps, paradoxical herniation, and external brain tamponade: a review of decompressive craniectomy management. Neurocrit Care.

[14] Yoshida K, Toda M, Yamada Y (2020). Sinking skin flap syndrome visualized by upright computed tomography. Acta Neurochir.

[15] Zhou L, Yu J, Sun L, Han Y, Wang G (2016). Overdrainage after ventriculoperitoneal shunting in a patient with a wide depressed skull bone defect: the effect of atmospheric pressure gradient. Int J Surg Case Rep.

[16] Holly LT, Batzdorf U (2001). Management of cerebellar ptosis following craniovertebral decompression for Chiari I malformation. J Neurosurg.

